# Discussing lifestyle behaviors: perspectives and experiences of general practitioners

**DOI:** 10.1080/21642850.2019.1648216

**Published:** 2019-08-05

**Authors:** Kyra Hamilton, Joanna Henderson, Emma Burton, Martin S. Hagger

**Affiliations:** a School of Applied Psychology and Menzies Health Institute Queensland, Griffith University, Brisbane, Australia; b School of Psychology, Health Psychology and Behavioral Medicine Research Group, Curtin University, Perth, Western Australia; c Psychological Sciences, University of California, Merced, CA, USA; d Faculty of Sport and Health Sciences, University of Jyväskylä, Jyväskylä, Finland

**Keywords:** General practitioners, general practice, lifestyle behaviors, health promotion, health communication

## Abstract

**Background:** Initiatives aimed at increasing participation in preventive health behaviors has been identified as a priority for addressing the increasing incidence of non-communicable chronic disease. General practice is an existing network that can be leveraged to intervene and promote messages for health behavior change. We aimed to explore the extent to which ‘lifestyle’ behaviors are discussed by general practitioners (GPs) with their patients in their practices, and the context and content of these discussions.

**Methods:** GPs (*N* = 26) practising in Australian clinics participated in semi-structured interviews. Data were analyzed using an inductive thematic analysis.

**Results:** Results showed discussions of lifestyle behaviors were brief, but relatively frequent and often initiated by the GP. GPs generally provided basic advice and education that was often ad-hoc and in reaction to prompts from the patient. GPs recognized the importance of addressing lifestyle behaviors in practice, but also highlighted substantive barriers that limit the initiation of these discussions. These included patient readiness for change, patient acceptance and openness, patient accountability and responsibility, patient background factors, GPs’ role and knowledge, GP financial implications, GP-patient relationship, and lack of time.

**Conclusions:** Current findings provide important preliminary knowledge on the extent to which Australian GPs discuss lifestyle behavior change with patients during routine consultations, the context and content of these discussions, and barriers to initiating these discussions. Further research should seek to gain a better understanding of barriers and identify strategies to mitigate their impact. This might maximize the potential for GPs to promote adaptive lifestyle behavior change for improving patient health.

## Introduction

Non-communicable diseases including heart disease, diabetes, and cancer are highly prevalent and make a substantive contribution to the global burden of morbidity and mortality, including in Australia (Australian Institute of Health and Welfare, 2016). These diseases are inextricably linked to ‘lifestyle’ behaviors such as physical inactivity, poor diet, smoking, and excessive alcohol consumption (Ford, Zhao, Tsai, & Li, 2011; Mokdad, Marks, Stroup, & Gerberding, 2004). The majority of primary health care in Australia is provided by general practitioners (GPs) (Australian Institute of Health and Welfare, 2016), with around 85% of the population visiting a GP at least once a year (Britt et al., 2015). In Australia, the Australian General Practice Training (AGPT) program provides training for registrars towards fellowship and gaining specialist (general practitioner) registration, which is usually three to four years of full-time training (Australian Government Department of Health, 2019). General practice is considered the cornerstone of primary health care in Australia, although primary health care can also include care provided through nurses (such as general practice nurses, community nurses and nurse practitioners), allied health professionals, midwives, pharmacists, dentists, and Aboriginal health workers (Australian Government Department of Health, 2018). Estimates demonstrate that among patients attending general practice in Australia, 61% are overweight, 17% are daily smokers, and 24% consume alcohol at risky levels (Britt et al., 2015); however, research indicates that GPs do not directly address these issues in consultations as a matter of routine (Brotons et al., 2012; Flocke & Stange, 2004; Kottke, Solberg, Brekke, Cabrera, & Marquez, 1997), a knowledge gap that needs to be addressed.

Studies conducted in the US and Europe have identified a number of factors as influencing lifestyle discussions in consultations, including GPs’ knowledge, self-confidence, perceived effectiveness of brief behavioral interventions, and limited time (Brotons et al., 2005; Thompson, 1996; Timmerman, Reifsnider, & Allan, 2000). Patients’ health literacy, readiness to change, and stigma have also been identified as influencing discussions (Beach, Roter, Wang, Duggan, & Cooper, 2006; Flocke, Kelly, & Highland, 2008; Mazza & Harris, 2010). Given GPs have contact with most of the Australian population every year, they are in an ideal position to raise and discuss lifestyle behaviors with their patients opportunistically during routine consultations, and often with positive outcomes (Ball, Johnson, Desbrow, & Leveritt, 2013; Ball, Lee, Ambrosini, Hamilton, & Tuffaha, 2016). For example, a systematic review of randomized controlled trials (Ball et al., 2013) showed improvements in the dietary behaviors of patients with lifestyle-related chronic diseases when brief nutrition care was incorporated into consultations, including observed improvements in a reduction of energy consumption of 0.7 MJ day–1, a reduction in excessive alcohol consumption of 36%, a reduction of meat consumption to three serves or less per week, a reduction of fat intake of 5–10%, an increase in fruit and vegetable intake by two serves per week, an increase in fish intake to at least one serve per week, and an increase in fiber intake of 0.55 g per 1000 kcal. GP advice has also been found to be effective in improving a range of other health behaviors including increasing physical activity (Dorsey & Songer, 2011; Elley, Kerse, Arroll, & Robinson, 2003) and smoking cessation (Stead, Bergson, & Lancaster, 2008). In addition, while a study conducted in the Netherlands found information provided by GPs was mainly given in generic terms (Noordman, Koopmans, Korevaar, van der Weijden, & van Dulmen, 2013), giving lifestyle advice that is tailored to the individual has been shown to be more effective than giving generic advice (Nobel, Paul, Carey, Blunden, & Turner, 2015). Although it should also be acknowledged that research, which was conducted in 22 European countries, found that a large proportion of patients attending primary care with unhealthy lifestyle behaviors did not believe they needed to change (Brotons et al., 2012).

Currently, there is some evidence that has explored how GPs discuss lifestyle behaviors with patients in routine consultations. The current study adds to this body of knowledge by exploring the extent to which ‘lifestyle’ behaviors are discussed by Australian GPs with their patients in their practices, and the context and content of these discussions. Findings extend previous research by providing important data on the adequacy of current provision for promoting preventive health behaviors and effective prevention in routine primary care, assist in identifying the important factors that lead to the initiation of these discussions, and provide an evidence base for future recommendations for opportunistic interventions delivered by GPs aimed at preventing chronic disease and promoting health.

## Materials and methods

### Participants

GPs practicing in the two Australian cities, Brisbane and Perth, were contacted through the professional networks of the research team and by referral from other participants. Consenting GPs (*N* = 26; females, *n* = 16, males, *n* = 10; Age M = 42.5 years, range 26–60 years) worked in practices catering for patients with private health insurance (*n* = 14) and government-supported Medicare insurance (*n* = 12). GPs had practiced for an average of 17 years (range 3–35 years). Twenty-two of the 26 GPs were registered GPs and four were GP registrars.

### Data collection

A semi-structured interview guide was developed and questions were informed by prior research and the clinical expertise of the research team. The interview guide was designed with the intention of gaining an in-depth understanding of the extent, context, and content of discussions regarding lifestyle behaviors between GPs and patients in general practice settings. The interview comprised four open-ended questions with additional probing questions to elicit further information (see Table 1). At the conclusion of the interview, GPs were invited to share any additional thoughts not raised in the course of the interview. Two final interviews were conducted to verify data saturation, which confirmed that additional data did not generate new themes.

**Table 1.** Overview of questions used to guide semi-structured interviews.

Research theme	Interview questions	Probing questions
Extent of discussions on ‘lifestyle’ behaviors	‘Thinking about the consultations with your patients, are health or lifestyle behaviors ever discussed?'; ‘To what extent do these discussions occur?'	How many patients they have these discussions with? Amount of consultation time used to have these discussions? Is there a formal process for these discussions (if they occur) or are they done on an ad hoc basis?
Context of discussions of ‘lifestyle’ behaviors	‘How do these discussions usually come about?'	Who initiates the discussion? Who contributions to the discussion? What is the context for the discussion (i.e. discussion came about through patients existing conditions, functional health concerns, weight loss or gain, pain, memory)?
Content of discussions of ‘lifestyle’ behaviors	‘What do you generally recommend in these discussions?'	What suggestions are made? What information given?

### Design and procedure

This study was approved by the institutional review boards at Griffith and Curtin Universities, Australia. The study adopted a qualitative research design using individual semi-structured interviews. Participants were sent invitations to participate via email or telephone, or in-person. Interviews were conducted in 2017 by two researcher assistants with a Bachelor of Psychology (Honors) degree and training in qualitative methods. Interviews were conducted via telephone or in-person at a location convenient to the participant and lasted approximately 30 min. Participants signed consent forms prior to data collection. A reflexive journal was kept by interviewers as a record of the key ideas expressed during the interviews, and to note comparisons and contrasts between the interviews (Braun & Clarke, 2006). Interviews were audio-recorded and transcribed verbatim.

### Analytic strategy

Inductive thematic analysis was used to analyze the data and identify patterns of meaning and themes (Braun & Clarke, 2006). This method was selected given its flexibility in regards to research questions, sample size, and method of data collection, and is not tied to a pre-existing theoretical framework. Data were analyzed according to the six phases outlined by Braun and Clark (Braun & Clarke, 2006). The first step involved familiarization with the data beginning with transcribing the interviews, reading and re-reading each transcript, and taking note of patterns and meanings relevant to the research questions. Next, a complete coding approach was taken which involved coding all extracts within the dataset that were relevant to the research questions using NVivo 11 by Author 2. Development of the coding-scheme was data-driven, with no codes specified a priori. To ensure the stability of coding and to enhance trustworthiness, Author 3 co-coded 10 interviews and all codes were reviewed by Author 1. Codes were collated inductively into potential themes, and all themes were reviewed with reference to the interview transcripts to ensure they reflected their original context. Themes were then reviewed, refined, and named by Author 1, 2, and 3. Finally, themes were reported and verbatim quotes that provided contextual significance were reported.

### Ethics Statement

All procedures performed in studies involving human participants were in accordance with the ethical standards of the institutional and/or national research committee and with the 1964 Helsinki declaration and its later amendments or comparable ethical standards.

## Results

Participants’ descriptions, and the themes that emerged from these descriptions, have been organized under the three main questions from the interview guide relating to the extent, context, and content of discussions regarding ‘lifestyle’ behaviors, and the influencing factors. Themes are presented alongside supporting quotes in Table 2, attributed to participants by their code number from the analysis (e.g. GP1). Next, we provide detail of emergent themes alongside commentary and interpretation arising from our analysis.

**Table 2.** Summary of key themes and supporting quotes from inductive thematic content analysis of interviews with general practitioners.

Discussion topic	Themes	Supporting quotes
Extent of lifestyle health discussions	‘Lifestyle’ behaviors discussed frequently but briefly	‘I would say the majority of consultations … more than fifty percent but not every single one' (GP1) ‘Oh well, it doesn’t take very long to say, I mean I just said it a minute ago and it took about 30 s, so I mean, 30 s out of an 8-minute consult maybe' (GP15) ‘Most of the time they haven’t come for a health behavior thing, so we sort of just quickly screen it … so that would be … like a one-minute thing … but then we would get them to come back to discuss that further and then we’d have a you know a fifteen minute-consult on the topic' (GP2) ‘If someone comes in for something really quick and easy and you deal with that, then I might make time to then deal with some preventative health thing that you might not always get time to do at other times' (GP2). ‘Well it’s based on what they present with mostly, if it is relevant to the situation or, the concern, so say, they are lacking in a behavior and it’s linked to their condition, um, well that is when I would raise the issue' (GP19). ‘It just … spontaneous and opportunistic … If age and other factors suggest it, it becomes opportunistic' (GP9)
	Recognising the importance	‘For a lot of things, it is really important … a lot of chronic health issues are well managed or better managed with … a healthy lifestyle' (GP8). ‘Realistically, you do know that the research shows if you put the effort in now, then five or ten years down the track, you won’t be dealing with so many issues, so it should be a factor in practise' (GP23). ‘We should do it more’ (GP10)
Context of lifestyle discussions	Patient prompts – Proactive and reactive	*Proactive ‘*There’s a much more broad sort of discussion of general health maintenance without there being a problem to solve, it’s more of a … ‘do you do any exercise?’, ‘do you do this or that?’ just from a general health promotion point of view rather than as a method for assisting with fixing something or maintaining health' (GP8) ‘Sometimes they’ll come in and ask, ‘look I want to have a general screen done’ or ‘I’m worried about my smoking, can you help me quit?’ or weight … let’s talk about that' (GP4). ‘Patients who say, “look I really wanna lose weight, can you tell me what to do?”' (GP10) ‘There are other cases where it is for regular review so for instance GP management plans we will bring them in for regular reviews for chronic disease such as diabetes’ (GP4) *Reactive ‘*If someone comes in and they’re obese you might make the time to talk to them about that' (GP1). ‘It is focussed on a presenting concern, or something related to that concern, I mean so if they have asthma and are a smoker, I will focus on that as my primary behavioral focus for the patient' (GP12). ‘Sometimes when they come in with a cold for instance I’ll ask if they have asthma or do they smoke and that will be the direction I go with the lifestyle question' (GP4) ‘It may well come up if you’re sort of aware that the patient’s demeanour is … not normal or has changed or … you’re aware of stressors in their life' (GP5) ‘I do a random sugar level … if that’s high that will be the instigator for the discussion' (GP4) ‘If you’ve got a young university student that’s stressed and needs a deferral for an exam before exams … I’m not going to mention it … I’m just going to do what they want me to do' (GP10). ‘In a general day to day, you know, script refills, um, maybe injuries or broken bones or, um yeah, then no I will not discuss it. It definitely depends on what is presented in the consultation and whether the discussion is appropriate’ (GP21). ‘If you come in with a rolled ankle you’d probably think it was a bit peculiar if I said … “are you depressed?” and they’d go “no I’ve just got a sore ankle”' (GP8)
	Prompts to cue discussions	‘There’s reminders that come up on our practice software. … smoking and alcohol we have prompts if it hasn’t been recorded … I think you can set it up to prompt for other things … then sometimes we act on that prompt … not always' (GP1) ‘I’d go through perhaps in a more structured way … their presenting complaint … background history, which would include smoking, alcohol, exercise perhaps not so specifically because there isn’t a particular … interface on our software for exercise' (GP7)
	GP-initiated or collaboratively-initiated	‘Most of the time it would be the doctor sort of flagging it and getting them to think about other aspects of their life that they might not even be aware of that are unhealthy' (GP2). ‘I would say usually it is me as a doctor bringing it up rather than the patient, unless say they have specifically come in for weight loss or a smoking cessation consultation, they would be the most health concerns most commonly brought up by my patients' (GP18) ‘Some people will come and initiate it and then they’ll say, ‘look I’ve been feeling really low’ … ‘I’m crying most days’ or ‘I’m drinking half a bottle … a bottle of wine a night … I know this is not right’ … they’ll come in and they recognise it' (GP4). ‘People who will come in and say … ‘I think I’m depressed’ … in which case they’ve initiated it … at other times … it’s much more of a physical presentation and then it’s more of us initiating to say, “look I think this is possibly what’s causing the issues here”' (GP8)
	Cost to GP vs. benefit to patient	‘It really is dependent on what other concerns need to be address and to be honest, how much time I have at the end to enquire about these things' (GP26) ‘I’m booked every ten minutes so if it’s outside of that time, because obviously if it’s a busy day and I’m just fully booked, then they may have to reschedule' (GP10). ‘I think it is really important, but I am struggling for time, I might approach it again at another consultation' (GP23) ‘If someone’s got ten other illnesses that are more pressing to them you’re not going to suddenly go ‘let’s talk about your smoking’ um but if there’s something mild and then you can see it’s still important and you can get them to see it’s important then zone in on it' (GP2) ‘Sometimes you just need to deal with a crisis' (GP5). ‘It depends on the urgency of those changes, for instance, if a woman is pregnant and smokes. … we kind of … a bit more pushier and prescriptive about those … but if this is a patient … who has been smoking and is about seventy years we are a bit more casual' (GP11)
Content of lifestyle discussions	Ad hoc with a lack of a formal process	‘It’s probably more ad hoc if it’s just an addition to the consultation … if it’s not related to their presenting complaint it’s probably a bit more ad hoc' (GP1). ‘Ad hoc if I’m trying to bring up the conversation with the patient so I will … jump at that opportunity' (GP4). ‘Basically, I will just bring it up and have a chat with them' (GP24). ‘What is appropriate information for the patient is something that, yes something that I generally do on the spot as the situation arises and the discussion progresses’ (GP17) ‘In my own mind it’s structured because you’re going almost through a tick box in your own mind of things … I don’t get out a sheet and say, “let’s go through this”' (GP8) ‘I don’t try and make anything too formal ‘cause I think it’s bad enough telling a stranger you’re depressed, you’re very vulnerable … and I think there’s still a lot of stigma about psychological stuff' (GP10)
	Content of discussion dependent on prompt or clinical indicator	‘Sometimes its [lifestyle discussions] part of the management of that particular presenting complaint' (GP1). ‘You kinda use that to fuel your argument for presenting why they should make a health behavior change' (GP2)
	Giving basic health advice and education	‘Well its really just information I have collated over the years, it is a combination of things that we have, say published referrals within the general practise, or that we have received from other help practitioners, also some national guidelines' (GP15). It’s very basic' (GP10) ‘As a brief intervention … eat less food, mainly plants … look at how much you drink alcohol and how much you move. So, move more, eat less … So, that’s the kind of brief things that I would say to most people' (GP3). ‘If they’re really depressed, really anxious … they’re unable to think you know they just can’t concentrate, they can’t focus, they can’t make a decision, so it’s quite important just to take that away from them and say, ‘look this is what you need to do, bang bang bang’, keep it simple … then always get them back' (GP6). ‘I don’t go into too much detail. I pretty much will let the psychologist deal with it all. I’m just there to sort of say look this is what I think you can probably look at doing’' (GP10). ‘I provide a lot of psychoeducation around the sort of symptoms around depression and anxiety and try to … get people to understand, especially with anxiety, the need to take some responsibility themselves' (GP7). ‘If it’s a young man who is in their forties and have risks … the person who has chest pain and all that … we use the fear factor as well as the persuasive method’ (GP9) ‘I certainly provide some education … in terms of the services … I’ll often give them an information leaflet about say depression, anxiety, stress, ways to cope etc … I’ll direct them to websites such as Beyondblue and Blackdog Institute … I suggest sometimes apps on phones so like relaxation or mindfulness apps like headspace' (GP4) ‘If we discuss a lot of issues and they sort of get out and go ‘oh my God, now what am I supposed to do?’ … So, I often write it down' (GP6) ‘It’s hard to know … which services are available, who to go to, what are the specialties of say a clinical psych' (GP4)
Factors influencing initiation of lifestyle discussions	Patient readiness for change	‘It all depends on their readiness for change … Sometimes it’s just really a matter of promoting the discussion for them to think about it ‘cause they’re not actually quite at that stage read to change things … So, I guess we’ve got to ascertain that first' (GP1). ‘The first question I ask is ‘do you want to quit? Are you at a stage where … ?’ and if they say no, it’s like ‘ok let’s talk about it again later’ … ‘cause what’s the point?' (GP10). ‘Um yeah I guess, yeah mostly it is just how easily the conversation flows and you know, if they look like, um, they are about to fall asleep' (GP19). ‘There’s no point if … they’re already frowning and shaking their head when you say the word ‘exercise’ then there’s no point going on for another ten minutes about it … you have to back off a bit' (GP8) ‘Sometimes you just give up ‘cause you know they’re not going to change … what do you do? What can you do?' (GP10). ‘It can be very difficult to make that a primary part of your practise when you have other acute medical conditions and the likelihood of change is slim' (GP25). ‘The patient has to have some sort of buy in or it is a waste of my time and theirs’ (GP15)
	Patient acceptance and openness	‘Patients that it’s more difficult raising lifestyle issues with … I guess they’re the ones that are in denial about their disease … trying to bring that up is a lot more difficult … it’s a … much more sensitive issue … so you just … you tread gently and just say ‘have you ever thought about’ … so you very gently sort of introduce those topics' (GP6) ‘Once the issue’s raised it’s very easy to engage … and get them to talk as well … I think it’s kind of like a vent for them … suddenly yes, someone’s going to listen to me' (GP2)
	Patient accountability and responsibility	‘People are responsible for their own health ultimately' (GP1). ‘There’s only so much you can do with adults you … try and empower them to sort of sort out things themselves … there’s only so much you can do … to get them to change things … they’ve gotta be doing some of this by themselves' (GP8). ‘I mean, they are adult, at the end of the day you can just give advice but really you cannot make them do anything that they don’t want to' (GP23)
	Patient background factors	‘The younger demographic they’re not usually concerned um and it’s more just about educating them' (GP2) ‘There’s sort of a background script but it’s very much tweaked upon what the patient already knows' (GP8) ‘They’re quite motivated cohort … they’re well educated, well-motivated, they understand the importance of good nutrition and good health and stuff and they’re always trying to optimise that I guess' (GP6) ‘They [men] are less likely to open up to someone that they don’t know … that’s a generalisation but I think that’s still true to an extent' (GP1). ‘I haven’t had too many males come in talking openly … or if they have … it’s because their wife has pushed theme' (GP4). ‘Men rarely like to come in … you feel like you have to mention what you can in the time ‘cause it’s the only chance they get' (GP3)
	GPs’ role and knowledge	‘I don’t go into detail because I don’t have the knowledge, so I tend to suggest go and see a dietitian … an exercise physiologist … because I don’t have enough knowledge to know what to do' (GP11). ‘I tend to refer because I’m not a psychologist … If I felt I had the knowledge, then I would deal with it’ (GP10) ‘It may be a … 45-year-old lady and you think ‘oh my God she’s carrying far too much weight’ but unless … she raises that herself … it would be very confronting to say' (GP2). ‘I’m not going to mention any of this [lifestyle behaviors] … I’m just going to do what they want me to do basically, because they’re not going to want me to be discussing how much exercise they do’ (GP10) ‘I think I haven’t actually done my job unless I deal with that and then say, ‘let’s have a look at your blood pressure’ … ‘what’s happening in your life at the moment?’ … find out what is happening for all of this person' (GP3)
	Financial implications	‘Health promotion, I mean is obviously an essential part of general practise, but that is not the area where you see the immediate rewards. So, there is human tendency to then not spend as much time, which is the nature of the beast' (GP20). ‘There is no time or monetary benefit for prevention' (GP19)
	GP-patient relationship	‘For me I feel you have to be careful not to nag people, you do want them to come back' (GP14) ‘If I haven’t met them before … I’d probably have to see them a few times to gauge ‘is this their normal?, or “have I noticed a trend in terms of their flat affect?”’ (GP4) ‘The more long-term the relationship is, the more likely people are perhaps to trust you with information' (GP7). ‘They’re usually pretty good about telling me you know whether they smoke a lot of week … whether they use meth or whatever but they’re often not the sort of things you can ask the first time you know it’s … the rapport' (GP3) ‘Just have to be fairly accepting, at least initially, so that people feel that they’re not going to be slammed down' (GP3). ‘I would probably hope that by talking about things that they would come back, again and again, and it would be something that you would be able to chip away at as your relationship builds' (GP18). ‘It comes down to a … trust thing … you gotta have a rapport with patients' (GP5)
	Lack of time	‘I think behavior change cannot be done in a fifteen-minute consult' (GP10) ‘They can get a lot of useful information [from the dietician] that I don’t necessarily have the time to … pick apart' (GP3) ‘I don’t go into detail ‘cause … basically I don’t have the time' (GP10). ‘Well yeah, I mean if they are looking at changing behavior, I suppose allied health, well they have the opportunities and resources to sit down with them and really focus on an in-depth plan, much more than what I could get across in the 2–3 min that I am talking to them' (GP24). ‘Behavior change cannot be done in a 10-minute consult' (GP10)

### Extent of lifestyle discussions

#### ‘Lifestyle’ behaviors discussed frequently but briefly

Most GPs stated lifestyle behaviors were discussed frequently but usually in brief. Patients would be required to attend a follow-up appointment to further discuss any lifestyle issues identified; however, no formal mechanism was cited to encourage follow-up. GPs commented that discussions regarding lifestyle generally occurred opportunistically.

#### Recognizing the importance

Most GPs recognized the importance in addressing lifestyle behaviors as part of routine consultations and noted their relevance to many health conditions. GPs admitted that more frequent discussions would be desirable and of longer duration.

### Context of lifestyle discussions

#### Patient prompts – proactive and reactive

Most GPs described that patient factors may prompt a proactive discussion of ‘lifestyle’ behaviors. GPs noted that they assessed smoking and drinking behaviors with all new patients and tended to raise other lifestyle behaviors when reviewing patients’ health records. GPs reported that lifestyle behaviors were commonly raised in response to the identification of predisposing factors such as for chronic disease from test results or the patient’s presenting complaint. Some GPs felt it inappropriate to address lifestyle if there was no overt prompt from the patient or relevant predisposing factor.

#### Prompts to cue discussions

A few GPs noted using practice software that prompted an assessment of lifestyle behaviors in new patients. Such prompts, however, needed to be manually programmed into the software and, unless GPs had sufficient knowledge and time to set up these alerts, it was unlikely that such prompts would be used to initiate lifestyle discussions in consultations. Discussions were, therefore, more likely to be raised in relation to other prompts or on an ad hoc basis rather than systematically as part of the consultation.

#### GP-initiated or collaboratively-initiated

Most GPs reported that patients sometimes initiated discussions of lifestyle behaviors. However, GPs acknowledged that these occasions were relatively rare, and they tended to initiate lifestyle discussions.

#### Cost to GP vs. benefits to patient

All GPs identified time as a key barrier to lifestyle discussions even though recognizing the potential benefits to patients’ long-term health. They also noted the need to prioritise patients’ most immediate problem, but also reported that in some cases it was important, or necessary, to initiate a discussion about lifestyle behaviors because it was integral to the presenting complaint.

### Content of lifestyle discussions

#### Ad hoc with a lack of a formal process

All GPs acknowledged that discussions of lifestyle behaviors were generally ad hoc and do not follow a formal, systematic process, with most arising in response to prompts from their examination of the patient or a test result, or in response to a prompt from the patient.

#### Content of discussion dependent on prompt or clinical indicator

Content of discussions was often context dependent; for example, based on an examination, test result, or routine screen. New patients, however, were often required to complete a comprehensive health assessment that included basic screening questions on health background, medical history, and, importantly, lifestyle behaviors such as smoking and alcohol intake. Some GPs also raised the importance of linking the patient’s immediate complaint with lifestyle behaviors where appropriate, and to discuss how the patient might make changes.

#### Giving basic health advice and education

All GPs indicated that the lifestyle behavior advice they provide is often basic, usually involving instructing patients on what behaviors they need to change, and messages on the consequences of not acting. Most GPs reported providing, or referring patients to, additional resources to complement the information provided, but also reported being unsure of the best resources and referral options to support patients’ lifestyle change.

### Factors influencing initiation **of lifestyle discussions**

#### Patient readiness for change

Patients’ degree of readiness for lifestyle behavior change was a factor that most GPs acknowledged impacted the initiation, and extent of, lifestyle behavior discussions, perceiving it futile to initiate, or dedicate time to, discussions if patients were not considered ready for change. Some GPs also acknowledged that some patients are unlikely to change, and that discussion or advice they gave would be unlikely to illicit change. However, there was no mention of how GPs typically assessed patients’ readiness to change, other than their cursory references to patients’ self-reported readiness gained from their brief discussions. None of the GPs mentioned formal assessment of patients’ readiness to change.

#### Patient acceptance and openness

Most GPs perceived patients’ openness to discussing lifestyle behaviors as an important factor influencing the initiation of lifestyle behavior discussions. Once a lifestyle behavior discussion had been initiated, GPs reported that most patients were open to further discussion.

#### Patient accountability and responsibility

All GPs were in agreement that patients should take responsibility for their health and be accountable for their lifestyle choices. GPs believed they needed to respect their patients’ choices, even if detrimental to health.

#### Patient background factors

Some GPs identified patient age as an important influence on the initiation of lifestyle discussions. GPs believed age would determine whether lifestyle discussions were initiated during routine consultations, conducting these less with younger samples, and also affect the content of the discussions, where more information was given to older samples usually because they had more health issues and chronic conditions to deal with. GPs also reported tailoring discussions based on patients’ health literacy. Some GPs indicated that patients with higher income and education usually meant they had greater health literacy and were more likely to proactively initiate lifestyle discussions. Some GPs reported that men tended not to openly discuss their issues, and also attended general practices less frequently than women. They therefore felt it more imperative to cover as much as possible in consultations with men.

#### GPs’ role and knowledge

Most GPs identified that their own level of knowledge had a significant influence on the lifestyle behavior discussions, acknowledging they did not have the necessary knowledge or skills to discuss lifestyle issues and make appropriate recommendations. They also expressed a level of conflict over their role during routine consultations; while GPs recognized that their primary role was to address patients’ current complaint and that it might be inappropriate to address other issues outside of this, they also held a belief that their duty of care should extend to promoting patients’ health beyond the current complaint.

#### Financial implications

It was evident that financial implications impacted GPs’ practice, and some admitted being reluctant to address certain issues with their patients if they were not remunerated. This was because such discussions take more time and ‘time is money’. For example, GPs reported there is an incentive to conduct in-depth health assessment for patients under the government-funded Chronic Disease Management plan, which enables GPs to plan and coordinate the health care of patients with chronic conditions. The plans meant that discussions and referrals regarding lifestyle change would be an integral part of consultations rather than ad hoc opportunistic discussions.

#### GP-patient relationship

Most GPs reported on the importance of the GP-patient relationship as an influencing factor on the initiation of lifestyle behavior discussions. GPs did not want to be perceived as ‘nagging’ or ‘preaching to’ their patients and believed that if they expressed their views too vociferously it might result in patient avoidance of future consultations or seeking out another practice. Having a good relationship and rapport with patients was discussed as a facilitating factor to raise lifestyle issues, as was being nonjudgmental or intrusive, techniques thought to increase the possibility that patients would be honest and open regarding their lifestyle behaviors.

#### Lack of time

All GPs referred to the impact of time on their discussions of lifestyle behaviors with patients. GPs reported that the length of lifestyle discussions often depended on time available and that lack of time was a barrier to initiating discussions. GPs reported that they would often refer patients to other health professionals because they did not have time to sufficiently assess and discuss lifestyle behaviors with patients during routine consultations. GPs also reported that they were generally able to provide only basic information to patients due to time pressures, and that specialist help on lifestyle change would be more effective.

## Discussion

The current study builds on existing knowledge by providing rich insights into the extent, context, and content of discussions on lifestyle behaviors by Australian GPs during routine consultations in general practice.

### Extent of lifestyle behavior discussions

Lifestyle discussions between GPs and patients in routine consultations were reported to be frequent, but brief, consistent with previous findings (Bardach & Schoenberg, 2014). GPs reported that when issues regarding lifestyle behaviors were flagged, patients would generally be asked to make a further appointment to discuss lifestyle issues, but also did not refer to any formal process to monitor whether patients made a follow-up appointment or any advocacy initiatives to encourage patients to return. This suggests that it may be beneficial for practices to implement reminder letter systems encouraging patients to return, a strategy known to be effective for increasing clinic attendance for preventive procedures such as screening (Task Force on Community Preventive Services, 2008).

### Context of lifestyle discussions

GPs recognized the importance of raising discussions of lifestyle with patients; however, concerns of being inappropriate in raising ‘other’ health issues if they were not directly relevant to the patient’s current complaint were also raised (Jacobsen, Rasmussen, Christensen, Engberg, & Lauritzen, 2005). Thus, GPs were more willing to have discussions about health behaviors with their patients if they were relevant to the presenting problem. For example, having a discussion around diet if blood tests came back indicative of pre-diabetes. This is consistent with a large study analyzing medical consultations collected between 1975 and 2008 in Dutch GP practices which showed GPs mainly refer to lifestyle when it is relevant to the patient's complaints (Noordman, Verhaak, & van Dulmen, 2010). Initiatives to allay GPs’ concerns over raising lifestyle behaviors might help to normalize preventive healthcare so that it becomes an excepted component of primary care. Current data also indicated that the responsibility for initiating lifestyle discussions often fall to the GP, and in response to prompts arising from routine examination or test results (e.g. blood screen). GPs reported that patients are often unaware or unwilling to proactively raise issues and seek help. Research conducted in the US has shown, however, that patients are often open to, and expect, information and advice regarding lifestyle behaviors from GPs (Kottke et al., 1997). Regularly discussing lifestyle in consultations may contribute to the overall perception that such discussions are part of routine care and may help increase patients’ knowledge and awareness of health issues and allay concerns over initiating lifestyle behavior discussions during GP visits.

Brief consultations require GPs to be optimally efficient in delivering care and prioritize the patients’ immediate concerns. GPs reported that discussing lifestyle behaviors is often not a priority given the limited time available in consultations and that time was insufficient to deliver effective advice. Given that GPs reported the need to account for all aspects of consultations, investigating how funding mechanisms may be modified to finance lifestyle behavior discussions in general practice is likely to be an important step. However, research suggests that financial incentives alone are less important than organizational factors. Healthcare practices with fewer patients and an electronic automated reminder system may enable more time for preventive care (Dahrouge et al., 2012). Encouraging people to attend routine health checks when they do not have a specific complaint may also increase GPs’ opportunities to raise lifestyle and preventive health. Such initiatives are consistent with research conducted in Australia showing strong links between health checks and preventive health behavior change (Amoroso et al., 2009). Involving practice nurses in preventive healthcare would also be of benefit, given GPs’ time constraints, as has been shown in the Netherlands (Voogdt-Pruis, Beusmans, Gorgels, Kester, & Van Ree, 2010).

### Content of lifestyle discussions

Discussions regarding lifestyle were reported to be ad hoc and unsystematic and often involved simple education. Thus, although GPs do discuss lifestyle behavior change as part of routine consultations, they do not do so in a consistent or systematic fashion. Research suggests that increasing motivation and readiness to change through health education is not sufficient to evoke changes in lifestyle behaviors (Coleman et al., 2012). This emphasizes the importance of GPs providing patients with more than just advice and suggests a need to better equip GPs with knowledge and skills to initiate discussions of lifestyle change and to motivate patients to adopt preventive health behaviors. GPs discussed arranging referrals to other health practitioners but expressed concerns regarding their lack of knowledge of referral pathways and available services. GPs would therefore benefit with having access to resources to assist with the referral process. The establishment of formal referral schemes may also assist, such as referral of individuals to identified pathways when specific indicators of disease risk arise in routine consultations, such as the UK GP referral scheme for physical activity (Williams, Hendry, France, Lewis, & Wilkinson, 2007).

### Factors influencing lifestyle discussions

Patient readiness to change was identified as an important factor impacting lifestyle discussions; however, GPs reported no formal means to assess patient readiness to change which may lead GPs to refrain from initiating lifestyle discussions. Providing GPs with tools to assess readiness to change may assist GPs in providing readiness-appropriate advice and referral (McConnaughy, Prochaska, & Velicer, 1983). Verheijden et al. (2005) suggest that including discussions of preventive health issues as part of routine primary care would lead to improved understanding of patient readiness to change and improve the delivery of advice relevant to patients’ level of readiness.

Patients’ openness and willingness to discussing lifestyle behaviors were also considered important. Furthermore, patients’ lack of understanding of preventive health and low levels health literacy were considered to impact understanding and thus embracing any advice given. A potential solution would be to recommend that GPs present discussions of lifestyle behaviors as part of routine consultations. This may help establish discussions as a norm and lead to patients being more open. Moreover, raising lifestyle behaviors in a climate of openness and in a non-judgemental fashion may reduce defensive reactions.

GPs recognized lack of knowledge and pathways for referral influenced whether or not lifestyle behavior discussions were raised during routine consultations and limited the extent of the advice provided when these issues were raised. This is consistent with research suggesting that health professionals’ self-efficacy in providing appropriate advice is a determinant of initiation of discussions on preventive health behavior. Research indicates that providing GPs with tools and support for a structured approach to behavior change may improve confidence in raising and managing lifestyle behaviors in the course of routine consultations (Ashman, Sturgiss, & Haesler, 2016). Furthermore, incorporating training in raising and delivering messages on lifestyle change to patients in consultations into GPs’ professional development may increase self-efficacy and motivation to incorporate preventive care in routine consultations (Amoroso et al., 2009).

### Study strengths and limitations

The current study has a number of strengths, including the adoption of a qualitative design which permitted in-depth discussion of lifestyle discussions initiated by GPs. Limitations should also be acknowledged. Most GPs interviewed were female. Female GPs have been shown to be more likely to provide counseling and advice than male GPs (Harrison, Britt, & Charles, 2011). Further, all GPs worked in metropolitan areas and, thus, not able to provide insight into how these issues are discussed in rural settings where health disparities are more prevalent and access to specialist healthcare is limited (Australian Institute of Health and Welfare, 2016). For example, previous research examining how GPs in rural areas discuss overweight and obesity with their patients has identified similar barriers to those identified in the current study (e.g. lack of time, concern about patient readiness) yet also identified unique barriers to the rural location (e.g. lack of referral options, impact of rurality on difficult conversations) (Glenister, Malatzky, & Wright, 2017). Interviews were also time limited due to the availability of GPs which may have restricted more descriptive views from participants being presented. Although current findings identified barriers that limit the extent and content of lifestyle discussions as part of routine consultations in general practice, future research should concentrate on better understanding these barriers and methods to overcome them. Finally, as with any study recruited through advertisements asking for volunteer participants, there is potential that GPs recruited to the current study were particularly motivated to provide lifestyle advice to patients, and current results should be interpreted in this light. However, the presence of these potential biases does not undermine the value of the contributions provided, particularly GPs comments on the challenges and barriers to providing lifestyle advice to patients.

### Conclusion

Current findings provide important preliminary knowledge on the extent to which Australian GPs discuss lifestyle behavior change with patients during routine consultations, the context and content of these discussions, and the barriers or enablers of these discussions. These data may provide evidence on which to base future research and, potentially, recommendations on how to effectively administer preventive care in general practice. GPs recognized the importance of addressing lifestyle behaviors in practice; however, substantive barriers exist that may limit the extent of these discussions and the potential of discussions to impact patients’ health. Further research should seek to gain a better understanding of barriers and identify strategies to mitigate their impact. Gaining this further knowledge might help to maximize the potential for GPs to promote adaptive lifestyle behavior change for improving patient health. Results have the potential to inform GP training and education in Australia, which could lead to improvements in the way lifestyle behaviors are discussed in general practice and offer the potential to develop opportunistic lifestyle behavior change interventions that may impact on health outcomes among at-risk populations.
